# Novel Spray Dried Algae-Rosemary Particles Attenuate Pollution-Induced Skin Damage

**DOI:** 10.3390/molecules26133781

**Published:** 2021-06-22

**Authors:** Roberta Hoskin, Erika Pambianchi, Alessandra Pecorelli, Mary Grace, Jean-Philippe Therrien, Giuseppe Valacchi, Mary Ann Lila

**Affiliations:** 1 North Carolina Research Campus, Plants for Human Health Institute, Food, Bioprocessing & Nutrition Sciences, North Carolina State University, Kannapolis, NC 28081, USA; rtcorrei@ncsu.edu (R.H.); mhgrace@ncsu.edu (M.G.); 2 North Carolina Research Campus, Plants for Human Health Institute, Animal Science, North Carolina State University, Kannapolis, NC 28081, USA; epambia@ncsu.edu (E.P.); apecore@ncsu.edu (A.P.); 3 JP Therrien Consulting, LLC, Davidson, NC 28036, USA; jptherrien@hotmail.com; 4 Department of Neuroscience and Rehabilitation, University of Ferrara, 44121 Ferrara, Italy; 5 Department of Food and Nutrition, Kyung Hee University, Seoul 02447, Korea

**Keywords:** cosmeceuticals, pollution, phytochemicals, herbs

## Abstract

The present study investigated the effect of spray-dried algae-rosemary particles against pollution-induced damage using ex-vivo human biopsies exposed to diesel engine exhaust (DEE). For this, the complexation of hydroalcoholic rosemary extract with Chlorella (RCH) and Spirulina (RSP) protein powders was conducted. The process efficiency and concentration of rosmarinic acid (RA), carnosic acid (CA), and carnosol (CR) phenolic compounds of both products were compared. The RSP spray-dried production was more efficient, and RSP particles presented higher CR and CA and similar RA concentrations. Therefore, spray-dried RSP particles were prioritized for the preparation of a gel formulation that was investigated for its ability to mitigate pollution-induced skin oxinflammatory responses. Taken altogether, our ex-vivo data clearly demonstrated the ability of RSP gel to prevent an oxinflammatory phenomenon in cutaneous tissue by decreasing the levels of 4-hydroxynonenal protein adducts (4HNE-PA) and active matrix metalloproteinase-9 (MMP-9) as well as by limiting the loss of filaggrin induced by DEE exposure. Our results suggest that the topical application of spirulina-rosemary gel is a good approach to prevent pollution-induced skin aging/damage.

## 1. Introduction

Striking evidence has demonstrated that air pollution can cause severe damage to human skin, triggering disorders such as inflammatory reactions, allergies, skin aging and cancer, due to alterations in physiological parameters that impact skin health [1,2,3,4]. In this regard, there is increasing consumer demand for cosmeceuticals based on natural products with the aim to improve skin beauty and protect skin from environmental insults [5,6,7,8]. A common pathway to identify promising sources of active phytochemicals involves the search for plant species traditionally used in folk or traditional medicine. For example, rosemary (*Rosmarinus officinalis*) is an aromatic plant that has long been used in herbal remedies due to its multiple biological activities including antioxidant, antimicrobial, anti-inflammatory [9,10,11] and skin renewal properties [12]. It is recognized as a major source of bioactives, mainly phenolic compounds such as rosmarinic acid, carnosic acid, and carnosol [13] and has attracted interest from food, pharmaceutical, and health-related industries worldwide [10,14]. In addition, algae-derived products are now part of a growing market, because algae can substitute for chemical and synthetic components in eco-friendly cosmeceuticals [15,16].

However, health-promoting phytochemicals are susceptible to thermal and oxidative degradation. Therefore, technological solutions are required to produce smart products with preserved natural phytoactives able to deliver active molecules in an easy-to-handle, stable format. In this context, we demonstrated that spray drying microencapsulation applied to the production of protein–polyphenol particles is an efficient tool to produce natural dried products with preserved characteristics and enhanced attributes [17,18,19]. This straightforward strategy complexes safe, wholesome proteins to a wide range of natural polyphenol-rich sources to create functional, clean-label products with concentrated levels of phytochemicals for multiple applications.

In this study, we developed spray-dried algae-rosemary particles using a hydroalcoholic rosemary extract complexed to algae-derived protein powders. Algae have recently been highlighted as natural rich sources of value-added metabolites with beneficial skin applications—in particular, Spirulina (*Arthrospira platensis*) and Chlorella (*Chlorella* sp.)-derived products [15]. Hence, by combining two recognized natural sources of metabolites with skin protective properties, our hypothesis is that we can achieve highly efficient formulations against pollution-induced skin damage. For this, spray-dried Spirulina-rosemary and Chlorella-rosemary particles were produced, and the process efficiency and concentration of rosmarinic acid, carnosic acid, and carnosol phenolic compounds of both products were compared. These two parameters were used as the selection criteria to prioritize a candidate experimental group that was used for the preparation of a gel formulation further investigated for its ability to mitigate pollution-induced skin oxinflammatory responses [20]. To the best of our knowledge, this is the first report that evaluates the effect of rosemary phenolics complexed to algae protein on the attenuation of pollution-induced skin damage.

## 2. Results

### 2.1. Solids Recovery and Characterization of Spray-Dried Algae-Rosemary Particles

**Figure 1:**
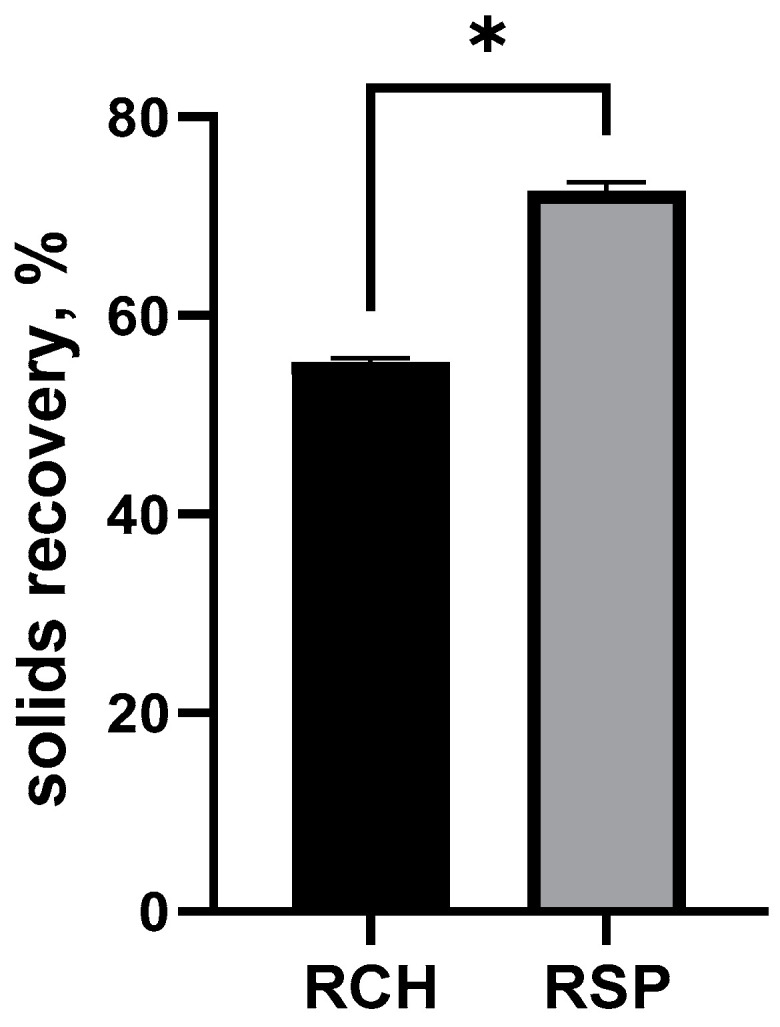
Solids recovery (%) of spray-dried algae-rosemary particles. RCH: Chlorella-rosemary particles and RSP: Spirulina-rosemary particles. Bars indicate standard deviation. Samples marked with an asterisk are significantly different: * *p* < 0.05.

Chlorella and Spirulina proteins were tested for their ability to mitigate spray drying stickiness and enhance production efficiency when complexed to hydroalcoholic rosemary extract. As shown in Figure 1, both experimental groups RCH and RSP achieved solids recovery higher than 50%, considered the threshold of successful spray drying operations [21]. However, the solids recovery of Spirulina-rosemary particles was higher (*p* < 0.05) when compared to Chlorella-rosemary spray drying process.

**Figure 2:**
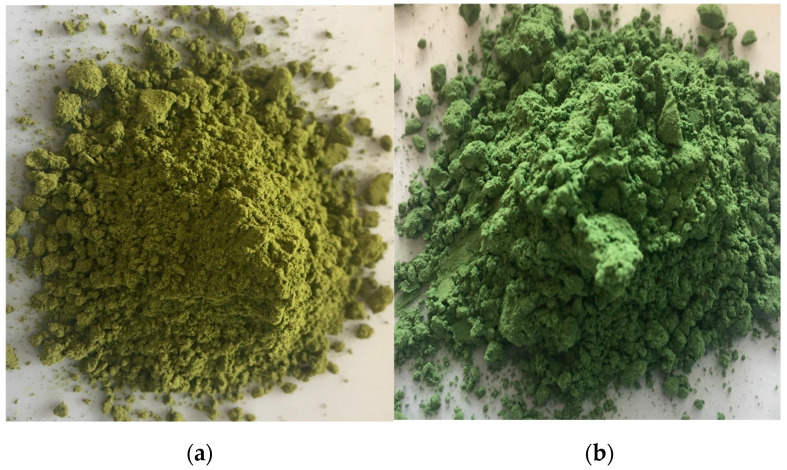
Visual aspect of spray-dried (**a**) Chlorella-rosemary (RCH) and (**b**) Spirulina-rosemary (RSP) particles.

RCH and RSP presented similar (*p* > 0.05) water activity (aw) levels (Table 1), which coincide with typical values of stable spray-dried products [22]. In addition, both RCH and RSP particles presented visually intense, attractive green color (Figure 2), confirmed by spectrophotometer color parameters that demonstrate samples with similar lightness L*, on the green side of the color spectrum (Table 1).

### 2.2. Concentrations of Rosmarinic Acid, Carnosol and Carnosic Acid

**Figure 3:**
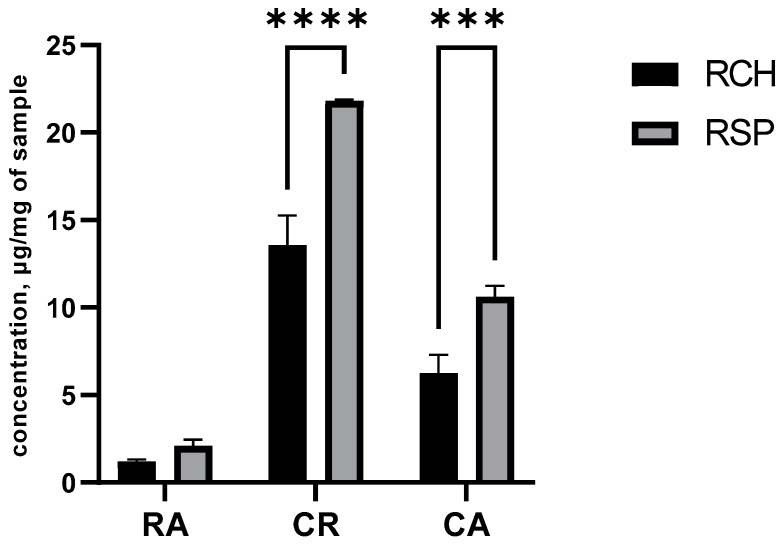
Concentrations of rosmarinic acid (RA), carnosol (CR) and carnosic acid (CA) in spray-dried algae-rosemary particles. RCH: Chlorella-rosemary particles and RSP: Spirulina-rosemary particles. Concentrations were calculated as μg/mg of spray-dried sample. Bars indicate standard deviation. Samples marked with asterisk are significantly different: *** *p* < 0.001; **** *p* < 0.0001.

Figure 3 shows that RSP particles captured significantly higher concentrations of carnosic acid and carnosol (*p* < 0.001), and a similar concentration of rosmarinic acid when compared to RCH particles. Thus, considering the increased solids recovery, similar RA and higher CR and CA concentrations, RSP was selected for gel formulation.

### 2.3. Evaluation of Pollution-Induced Skin Damage

**Figure 4:**
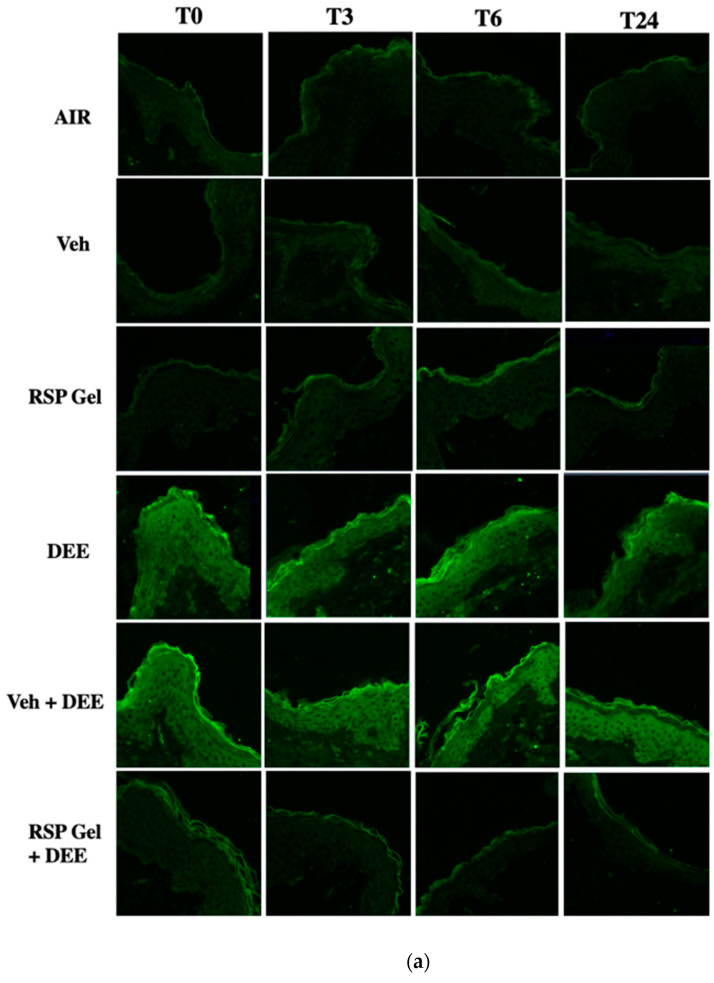

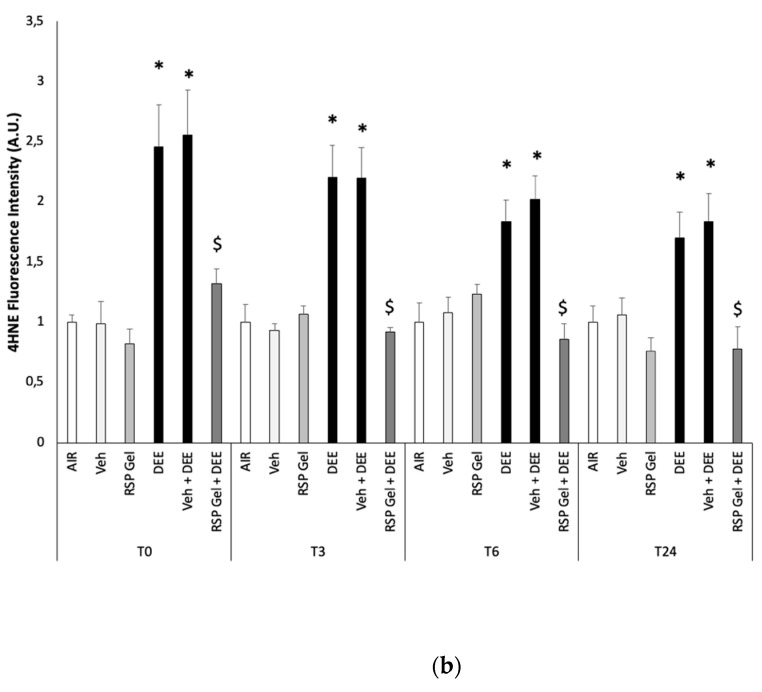
Skin exposure to DEE increases protein damage mediated by lipid peroxidation (i.e., 4HNE), while topical application of RSP gel prevents this effect. Levels of 4HNE-PA in ex vivo human skin explants untreated or pre-treated with RSP gel (**a**) Green fluorescence staining represents 4HNE-PA immunoreactivity. Original magnification 40×. (**b**) Semi-quantification of the immunofluorescence intensities performed by ImageJ is shown in the histograms. Data are expressed as arbitrary units (averages of three independent experiments), * *p* ≤ 0.05 Air vs DEE or Veh + DEE; $ *p* ≤ 0.05 DEE or Veh + DEE vs RSP Gel + DEE, by ANOVA.

As depicted by the immunofluorescence assay in Figure 4a, DEE significantly promoted the formation of cutaneous 4HNE-PA, a well-known marker of lipid peroxidation-mediated protein damage [23]. In particular, the 4HNE-PA signal was evident in the stratum corneum and epidermis, which are the main targets of pollution-mediated damage [24]. Of note, 4HNE-PA immunofluorescent reaction was also slightly detected in the dermal layer of DEE-exposed skin (Figure 4a). Quantification of fluorescence intensity confirmed the increase of 4HNE-PA levels after DEE exposure (T0, 30′) and, although with a declining trend, remained significantly higher in pollutant-exposed tissues than in air-exposed skin for up to 24 h after DEE insult (Figure 4b). The topical pretreatment for 24 h with RSP gel significantly prevented 4HNE-PA formation in DEE-exposed skin tissues, to a similar extent for all time points tested (Figure 4b). No effect of RSP gel and vehicle (Veh) was noticed in air-exposed skin explants (Figure 4a,b).

**Figure 5:**
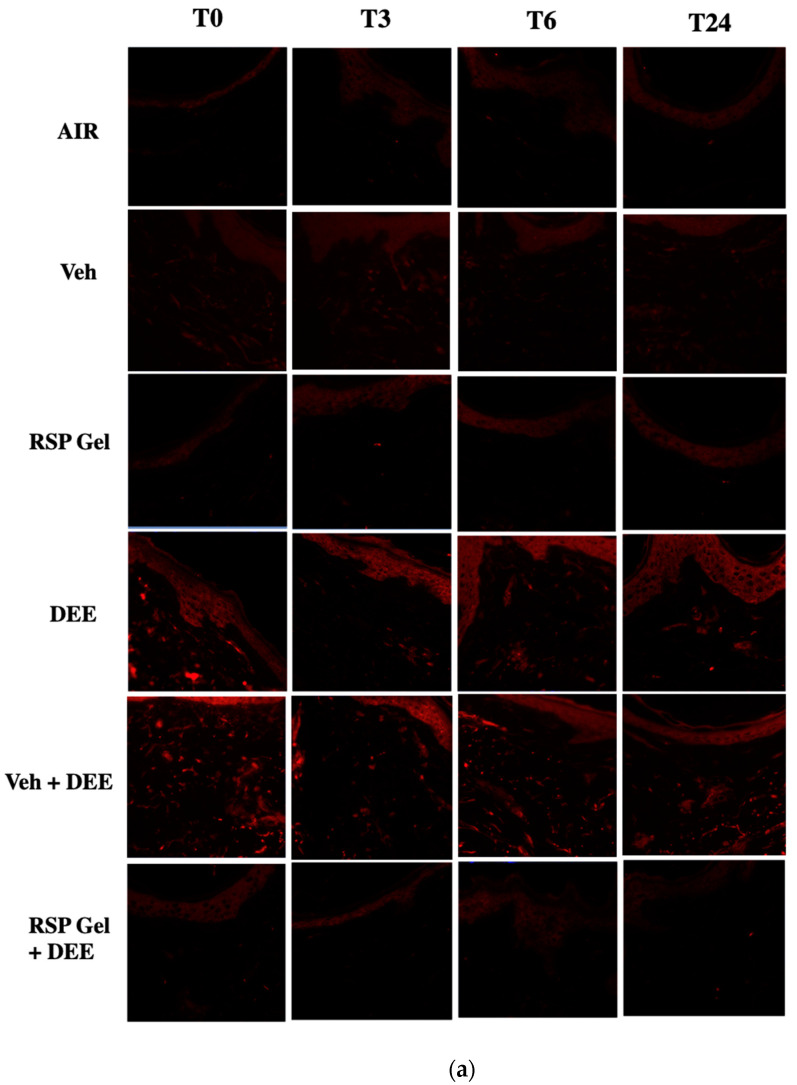

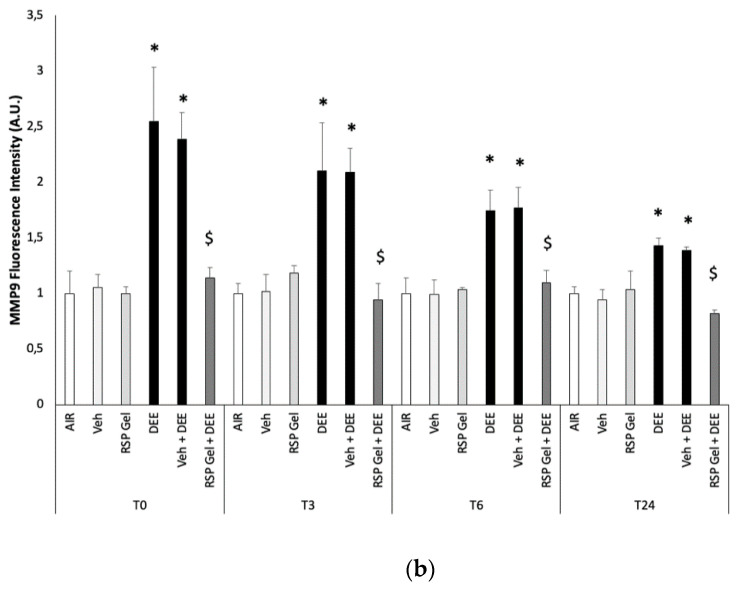
Skin exposure to DEE increases the levels of matrix metalloproteinase-9 (MMP-9), while topical application of RSP gel prevents this effect. Levels of MMP-9 in ex vivo human skin explants untreated or pre-treated with RSP gel (**a**) Red fluorescence staining represents MMP-9 immunoreactivity. Original magnification 40×. (**b**) Semi-quantification of the immunofluorescence intensities performed by ImageJ is shown in the histograms. Data are expressed as arbitrary units (averages of three independent experiments), * *p* ≤ 0.05 Air vs DEE or Veh + DEE; $ *p* ≤ 0.05 DEE or Veh + DEE vs RSP Gel + DEE, by ANOVA.

Accumulation of 4-HNE-modified proteins in the skin tissue can trigger cascades of proinflammatory signals and mediators including extracellular matrix-degrading metalloproteinases (MMPs) [25]. Therefore, we next evaluated the activation of MMP-9 by DEE exposure in the presence or not of the RSP gel. As depicted in Figure 5a, the dermal layer of DEE-exposed skin showed a higher immunoreaction for the active form of MMP-9 than that observed in skin tissues not exposed to DEE. The quantification of fluorescence signal demonstrated that the increase of MMP-9 active form was more evident 30 min after DEE exposure, and its increase was still significant at 3, 6 and 24 h (Figure 5b). As observed for 4HNE-PA, 24 h pretreatment with algae-rosemary gel was able to inhibit the increase in active MMP-9 levels in skin tissues exposed to DEE (Figure 5a,b). No effect of the vehicle (Veh) only treated tissues was noticed.

**Figure 6:**
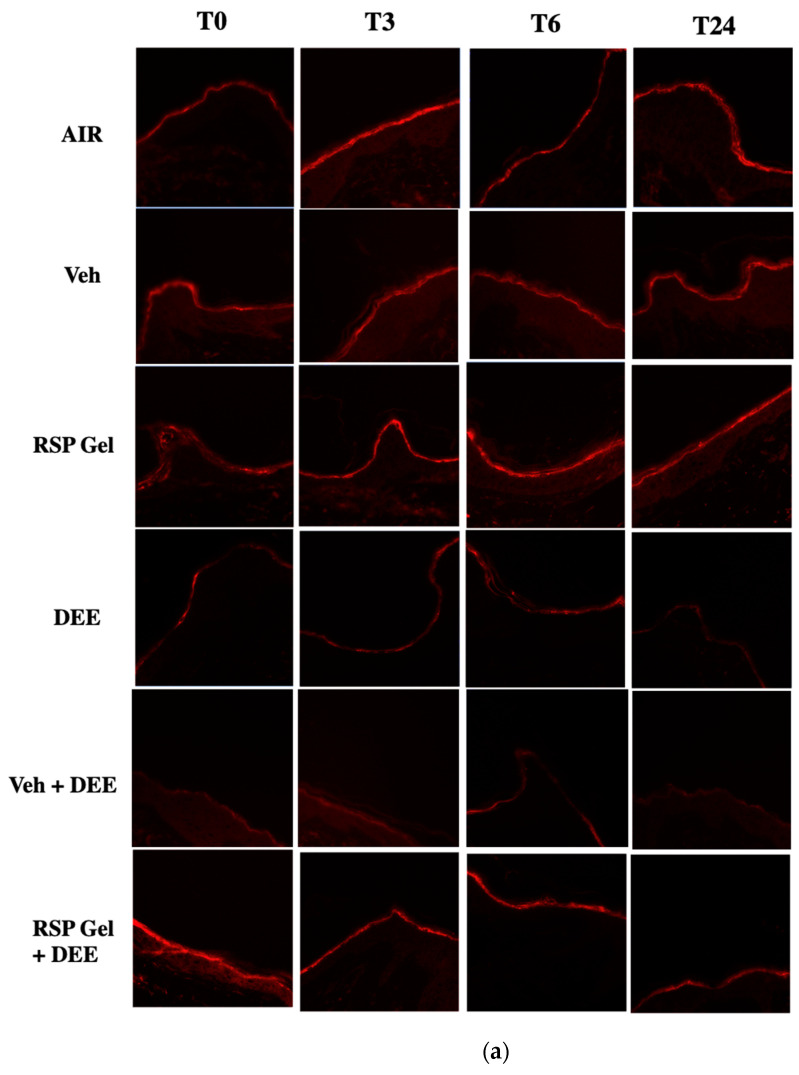

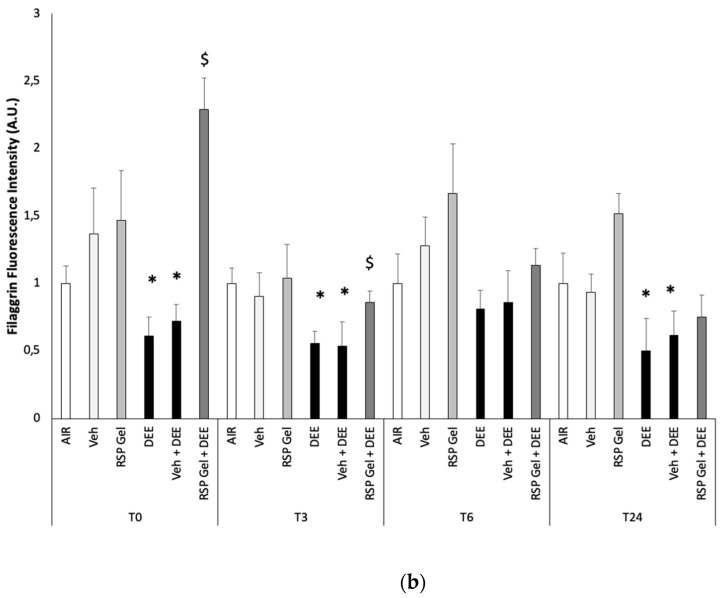
Skin exposure to DEE decreases filaggrin levels, while topical application of RSP gel prevents this effect. Levels of filaggrin in ex vivo human skin explants untreated or pre-treated with RSP gel (**a**) Red fluorescence staining represents filaggrin immunoreactivity. Original magnification 40×. (**b**) Semi-quantification of the immunofluorescence intensities performed by ImageJ is shown in the histograms. Data are expressed as arbitrary units (averages of three independent experiments), * *p* ≤ 0.05 Air vs DEE or Veh + DEE; $ *p* ≤ 0.05 DEE or Veh + DEE vs RSP Gel + DEE, by ANOVA.

Skin exposure to pollution can also impact cutaneous barrier functions [26]. As shown in Figure 6a, DEE exposure clearly decreased the filaggrin levels (a key protein involved in maintaining the stratum corneum properties). Indeed, we observed a clear decrease in immunofluorescence staining for filaggrin in the stratum corneum of DEE-exposed skin (Figure 6a), and this was evident already 30 min after DEE exposure and persist also after 3 and 24 h post-exposure (Figure 6b). Notably, 24 h-pretreatment with RSP gel prevented the stressor-mediated decline in filaggrin levels (Figure 6a,b).

## 3. Discussion

Spray drying is extensively used in the food and pharmaceutical industries to produce plant-derived products in a scalable, cost-effective manner. It presents several advantages such as short drying time, lower electricity consumption, and allows the production of regularly shaped particles obtained in a single-step procedure [27]. The solids recovery (%) is an important spray drying parameter defined as the relationship between the solids content of the final powder and the initial solid content in the feed mixture. This parameter can be affected by heat-induced reactions that lead to stickiness and consequent particle adhesion to chamber walls, causing product losses and drying difficulties. In other words, higher solids recovery translates into more powder and increased efficiency, and for this reason, it is frequently referred to as drying efficiency or drying yield [22].

One of the main factors affecting drying efficiency is the type and concentration of wall material [28]. Indeed, because proteins are efficient film-forming molecules with important surfactant activity, they preferentially migrate to the air–water interface and envelope bioactive compounds, forming a physical barrier that enables higher drying efficiency [29]. In this study, we showed that the solids recovery of Spirulina-rosemary particle production was higher (*p* < 0.05) when compared to Chlorella-rosemary spray drying process. This higher recovery might result from enhanced surface-active properties and film-forming capacity caused by the higher protein content of Spirulina protein powder used in this study [30,31].

Water activity is an important attribute of powdered products since it dictates the storage stability and susceptibility to degradation reactions [32]. Both RCH and RSP presented similar (*p* < 0.05) aw levels within the range typically found for stable spray-dried powders [33], besides displaying attractive, vibrant green colors (Figure 2). The spray dried algae-rosemary particles were also characterized regarding the concentration of rosemary phenolic diterpenes, carnosic acid (CA), carnosol (CR), and rosmarinic acid, recognized as potent antioxidant molecules with superior performance when compared to synthetic antioxidants used in the industry [9] and several biological activities [14]. Our results (Figure 3) show that RSP particles captured significantly higher concentrations of rosemary antioxidants when compared to RCH particles. We previously demonstrated that protein forms a protective layer during spray drying that may serve as a protective shell around core phytocompounds and avoids potential degradation during drying and storage [17,19]. In this case, spirulina protein powder exerted a more efficient role and therefore, this group was selected for further pollution-induced damage evaluation using human skin biopsies.

In particular, to explore the potential efficacy of the RSP formulation in modulating an oxinflammatory event, i.e., a phenomenon characterized by a harmful interplay between an imbalance redox homeostasis and a subclinical inflammation [26], a gel formulation containing 100 µg/mL RSP particles was tested in an ex vivo human skin model exposed to DEE. Taken altogether, our ex vivo data clearly demonstrated the ability of RSP gel to prevent pollution-induced 4HNE-mediated protein damage, extracellular matrix degradation and epidermal barrier dysfunction in cutaneous tissue. Several mechanisms could underlie these protective effects, mainly related to the high concentration of carnosic acid and carnosol present in the Spirulina-rosemary gel tested in this study. In fact, in vitro experimental results revealed the ability of both carnosic acid and carnosol of preventing lipid oxidation through different processes [34]. For example, carnosic acid directly quenches reactive pro-oxidant compounds, whereas carnosol is resistant to oxidation by reactive oxygen species (ROS), but has an inhibitory effect on the lipid peroxidation process by reacting directly with lipid radicals [34]. Furthermore, in different cell types including neuronal and hepatic cell lines, rosemary extract and its polyphenolic diterpenes demonstrated to activate the Nrf2 antioxidant response pathway by inducing the transcription and expression of several cytoprotective enzymes such as NAD(P)H: quinone oxidoreductase 1 (NQO1) and hemeoxygenase-1 (HO1) [35,36,37,38]. Therefore, our hypothesis is that the cutaneous topical application of RSP gel was able to prevent DEE-induced 4HNE-PA formation through the combination of both mechanisms. On one hand, carnosic acid and carnosol can directly quench reactive oxidants and limited lipid peroxidation reactions and, thus, 4HNE generation during DEE exposure. Secondly, the pretreatment with RSP gel for 24 h may have induced the activation of the Nrf2-mediated antioxidant response, boosting the cutaneous defensive antioxidant enzymes. In line with this hypothesis, it was demonstrated the hormetic effects of carnosic acid and carnosol in inducing an Nrf2-dependent antioxidant defense in normal human skin fibroblasts. The phenolic diterpenes exhibited potential antiaging effects in human skin fibroblasts by preventing hydrogen peroxide-induced premature senescence. In addition, carnosol ameliorated several features of cells undergoing replicative senescence (i.e., aging) [39].

Moreover, carnosic acid was effective against deleterious effects of pollutants including urban dust and cigarette smoke extract in normal human skin fibroblasts and epidermal keratinocytes. In particular, carnosic acid induced a significant increase in HO1 and NQO1 gene expression in normal human skin fibroblasts exposed to urban dust, thus enhancing the cellular endogenous defenses against an oxidative insult. In addition, carnosic acid significantly prevented the MMP-1 mRNA upregulation induced by cigarette smoke extract in normal human skin fibroblasts [40]. Recently, the inhibition of TNFα-induced MMP-1 secretion has also been attested in dermal fibroblasts treated either with carnosic acid or rosemary extract [41]. Similarly, pretreatment with carnosic acid inhibited expression and release of multiple MMPs induced by ultraviolet irradiation in both primary human dermal fibroblasts and primary human epidermal keratinocytes, demonstrating that carnosic acid can counteract UV-induced skin photoaging [42].

Consistently with previous findings, our study indicated the ability of algae-rosemary gel to inhibit the increase in active MMP-9 levels induced by DEE exposure in the skin tissue. These effects could be related to the well-recognized immunomodulatory functions of rosemary extract and its active constituents [43]. Specifically, the mechanism by which RSP gel ameliorated the DEE-induced skin inflammation could be explained through the inhibition of the nuclear factor kappa B (NF-κB) signaling pathway. Indeed, in a dose-dependent manner, carnosol inhibited IL-1β-stimulated nuclear translocation of NF-κB-p65, decreasing the gene expression of inflammatory genes in human chondrocytes [44]. Another study showed similar results in monocyte/macrophage-like cells, confirming the ability of a methanol extract of rosemary to inhibit LPS-induced MAPKs and NF-kB activation [45].

This effect could also justify our results on the filaggrin expression. Indeed, a downregulated expression of filaggrin was also observed in human keratinocytes exposed to urban particulate matter and the underlying mechanisms involved ROS-mediated activation of the transcription factors NF-κB and AP-1 [46]. Therefore, the combined ability of algae-rosemary gel to limit the 4HNE-mediated protein damage through a probable enhancement of the skin defense functions and to restrain the DEE-stimulated inflammatory state by modulating the NF-kB signaling pathway could contribute to its protective effects against DEE-induced loss of filaggrin, as already demonstrated for another plant-derived phenolic compound [47]. In addition, it should be mentioned that the loss of filaggrin could not only affect skin barrier functions and possibly promote skin conditions related to pollution exposure, but it could even facilitate the entrance of exogenous pathogens and allergens that can further damage the cutaneous tissues [48,49]. Finally, it should be mentioned that the present study was performed in an “ex vivo” model, which, although is one of the most reliable approaches to represent skin tissue in a laboratory, it is still a model that has some limitations. One impediment of existing ex vivo tissue culture systems is that viability and integrity of the tissue can generally not be maintained for a long period; in addition, the lack of circulation and innervation, which are present in in vivo models, preclude the possibility to evaluate a complete inflammatory response. Therefore, these aspects need to be considered before translating ex vivo results to human subjects.

## 4. Materials and Methods

### 4.1. Chemicals

All organic solvents were HPLC grade and obtained from VWR International (Suwanee, GA, USA). Organic Chlorella protein powder (50% protein, Triquetra Health, Tampa FL, USA), organic Spirulina protein powder (66% protein, Zazzee Naturals, Austin, TX, USA) and xanthan gum (MakingCosmetics, Snoqualmie, WA, USA) were used in this study.

### 4.2. Preparation of Rosemary Hydroalcoholic Extract

Fresh rosemary shoots with leaves (*Rosmarinus officinalis*, variety Spice Mountain) were locally obtained (John Weddington Greenhouse, Salisbury, NC, USA). Fresh leaves (moisture content 71.3% ± 2.0%) were stripped off and extracted with aqueous ethanol under vacuum to minimize the oxidation of phenolic constituents. Batches of 200 g were processed for 15 min using a blender (Vita-Mix Corp, Cleveland, OH, USA) attached to a vacuum accessory. The mixture was sonicated for 15 min and the supernatant was collected and centrifuged at 4 °C. The rosemary solid fraction was re-extracted with 500 mL of 80% aqueous ethanol using a similar protocol, followed by a third extraction using 500 mL of 50% aqueous ethanol. The combined supernatant (approx. 2.5 L) was vacuum evaporated to remove ethanol. A final hydroalcoholic rosemary extract containing 7.3 ± 0.2% dry matter was obtained (final volume of approx. 400 mL). Several batches extracted over a period of 48 h were kept under refrigeration at 4 °C and mixed to form one single batch used for all spray drying experiments.

### 4.3. Spray Drying

Two experimental algae-rosemary groups were investigated in this study: rosemary extract complexed with organic Chlorella protein powder (RCH) and rosemary extract complexed with organic Spirulina protein powder (RSP). Before each production batch, the algae protein powders were dispersed directly into the hydroalcoholic rosemary extract by vigorous mixing (PRO Scientific Bio-Gen PRO200, Oxford, CT, USA) for 2 min at 15,000 rpm until complete dissolution. The prepared feed solution was atomized using a spray dryer (B-290, Buchi Labortechnik AG, Switzerland) at 110 °C (outlet temperature 58–61 °C) following preliminary experiments (data not shown). The spray drying system operated using air in co-current flow under the following optimized conditions: 1.5 mm diameter nozzle, 10 mL/min of feed flow (controlled by peristaltic pump) kept under constant magnetic stirring during drying. For each algae-rosemary sample, 10% (*w*/*v*) of algae protein powder was added to the hydroalcoholic rosemary extract. The resulting spray-dried particles were collected from the collection chamber only, and all the other particles adhering to the drier walls and/or pipes were discarded. The final powdered product was weighed and immediately sealed in Ziploc^®^ bags. The solids recovery (also referred to as production yield) of spray-dried algae-rosemary samples was calculated as a percentage (%) according to the ratio [total solids content of resulting particles (algae-rosemary particles)/total solids content in the feeding mixture (before spray drying)] × 100 according to our previous protocol [17].

### 4.4. Water Activity and Color Measurements

The water activity of spray-dried algae-rosemary samples was measured using an Aqualab water activity meter (Decagon, Pullman, WA, USA). A reflectance spectrophotometer (CR-400, Konica, Minolta, Japan) previously calibrated with white and black standards was used to determine the color parameters lightness (L*), greenness (−a*) or redness (+a*), and blueness (−b*) or yellowness (+b*).

### 4.5. HPLC-DAD Analysis for Rosmarinic Acid, Carnosol, and Carnosic Acid

HPLC-DAD was conducted on an Agilent 1200 series HPLC (Agilent Technologies, Santa Clara, CA, USA) equipped with a photodiode array detector (DAD) set at 230 nm. The chromatographic separation was performed on Phenomenex Synergi 4 μm hydro-RP 80A column (250 mm × 4.6 mm × 5 μm, Torrance, CA, USA) thermostatted at 25 °C according to our previously described method [50]. Rosmarinic acid (RA), carnosol (CR) and carnosic acid (CA) were quantified based on standard curves constructed with corresponding reference standards and expressed as μg/mg DW.

### 4.6. Preparation of Gel Samples Containing Algae-Rosemary Particles

Initially, a solution of 0.25% (*w*/*v*) of xanthan gum was prepared and mixed for 30 min using a magnetic stirrer until complete dissolution. This xanthan gum gel was used to prepare a hydrocolloidal solution containing 100 µg/mL of Spirulina-rosemary particles (selected in the first part of the study) that was used for all skin tissue experiments.

### 4.7. Ex Vivo Human Biopsies Treatment and Exposure to Diesel Engine Exhaust (DEE)

Healthy human skin, purchased from a local hospital (Hunstad/Kortesis/Bharti Cosmetic Surgery clinic), was obtained from three different donors undergoing elective abdominoplasty, as approved by the IBC at NC State University (USA). Subcutaneous fat was trimmed from 12 mm punch biopsies, and biopsies were rinsed with PBS containing antibiotics/antimycotic. Next, biopsies were cultured in DMEM containing 10% FBS, 100 U/mL penicillin, and 100 ug/mL streptomycin at 37 °C in 5% CO_2_ with the upper part of the epidermis exposed to the outside environment.

The next day, medium was changed, and the algae-rosemary gel (RSP) or the only vehicle (Veh) (xantan guam 0.25%; *w*/*v* solution) were topically applied. After 24 h of pre-treatment, biopsies were exposed for 30 min to DEE generated by a Kubota RTV-X900 diesel engine (3-cylinder, 4-cycle diesel with overhead valves, 1123 cc that has 24.8 HP at 3000 rpm) and cultured in fresh medium for the different time points (3, 6 and 24 h) following the DEE exposure.

### 4.8. Immunofluorescence

Paraffin-embedded 4 µm sections of skin biopsies were deparaffinized in xylene and rehydrated in decreasing alcohol gradients. Antigen retrieval was achieved using heat-based epitope retrieval with sodium citrate buffer (Thermo Fisher Scientific, Waltham, MA, USA) (pH 6.0) at a sub-boiling temperature in a 500-watt microwave for 10 min. After cooling, sections were washed in PBS, blocked with 5% BSA in PBS, and incubated with primary antibodies for 4HNE (dil. 1:400; AB5605, Millipore), MMP9 (dil. 1:200; NBP2-13173, Novus Biologicals), filaggrin (dil. 1:50; sc-66192, Santa Cruz Biotechnology, Inc.), in 2% BSA in PBS. Sections were then washed in PBS and incubated with fluorochrome-conjugated secondary antibodies (dil. 1:1,000) (Alexa Fluor 568 A11004 or Alexa Fluor 488 A11055) in 2% BSA in PBS at RT, and then washed with PBS. Nuclei were stained with DAPI (1874814, Invitrogen) in PBS, and sections were then washed with PBS. Sections were mounted using PermaFluor mounting medium (Thermo Fisher Scientific) and imaged on a Zeiss LSM10 microscope. Images were quantified using ImageJ.

### 4.9. Statistics

Statistical analyses were performed using GraphPad Prism 6 software (GraphPad Software Inc., La Jolla, CA, USA). For comparisons between groups, analysis of variance (ANOVA) followed by Bonferroni’s post-hoc test was conducted. All data were expressed as means ± standard deviations and statistical differences between means were determined using *p* ≤ 0.05 significance level.

## 5. Conclusions

In this study, we investigated a strategy to take advantage of phenolic-rich hydroalcoholic rosemary extract complexed with algae-derived protein for skin health applications. The present study demonstrates the protective effect of these novel spray-dried algae-rosemary particles against one of the most noxious pollutants, diesel exhaust, to which our skin is daily exposed. Collectively, our results suggest that the topical strategy using rosemary–spirulina gel is a good approach to prevent pollution-induced skin aging/damage.

## Figures and Tables

**Table 1:** Water activity and color parameters of spray-dried algae-rosemary particles ^1^.

	RCH	RSP
Water activity	0.323 ± 0.001	0.314 ± 0.001
L*	50.12 ± 1.54	50.96 ± 0.31
a*	−5.93 ± 0.63 ^b^	−7.34 ± 0.06 ^a^
b*	14.73 ± 1.55 ^a^	8.42 ± 0.10 ^b^

^1^ RCH: spray-dried Chlorella-rosemary particles and RSP: spray-dried Spirulina-rosemary particles. Superscripts with different letters (^a,b^) in the same row are significantly different (*p* < 0.05). Results are shown as mean ± standard deviation. CIELAB parameters: L*: lightness; color coordinates: a*—green to red; b*—blue to yellow.

## Data Availability

All data generated or analyzed during this study are included in this published article.
